# Unveiling the hidden world of microorganisms and their impact on the Earth's ecosystems

**DOI:** 10.1002/mlf2.12100

**Published:** 2023-12-29

**Authors:** Yunfeng Yang, Jizhong Zhou

**Affiliations:** ^1^ Institute of Environment and Ecology, Tsinghua Shenzhen International Graduate School, Tsinghua University Shenzhen China; ^2^ Institute for Environmental Genomics University of Oklahoma Norman Oklahoma USA; ^3^ School of Biological Sciences University of Oklahoma Norman Oklahoma USA; ^4^ School of Civil Engineering and Environmental Sciences University of Oklahoma Norman Oklahoma USA; ^5^ School of Computer Sciences University of Oklahoma Norman Oklahoma USA; ^6^ Earth and Environmental Sciences Lawrence Berkeley National Laboratory Berkeley Califonia USA

Microorganisms are the unseen drivers of the Earth, impacting everything from the air we breathe to the soil beneath our feet. The roles they play in maintaining ecosystem functioning, particularly in the biogeochemical cycling of carbon, nitrogen, phosphorus, sulfur, and metals, are profound. Microbial communities are critical in mediating organic carbon mineralization, methane cycling, denitrification, and sulfate reduction, all of which regulate carbon storage and greenhouse gas emissions.

Microbial ecology has made significant advancements since the 1970s, transitioning from a culture‐based microbial ecology in the 1980s to a genome‐based microbial ecology after the 2000s. This shift became possible thanks to pioneering work from Dr. James M. Tiedje and other leading microbial ecologists^1^. To date, the use of cutting‐edge experimental and computational technologies has started to illuminate the hidden world of these microorganisms, providing an avenue to explore microbiome functions in diverse environments such as the ocean, soil, and wetlands. This has significantly broadened our understanding, enabling us to investigate the functional potential of microbiomes and understand their influence on the environment.

To celebrate James M. Tiedje's 80th birthday and honor his outstanding contributions to microbial ecology, the Institute for Environmental Genomics at the University of Oklahoma hosted a special symposium in association with iFAST (International Forum on Advanced Environmental Sciences and Technology), which is an interactive online forum for eminent scientists to share their most recent advances in environmental sciences and technology with and foster interdisciplinary networking among environmental researchers, engineers, students, and the general audience. The iFAST seminars have covered a diverse array of frontier research topics in environmental sciences and technology, particularly those related to climate change, environmental protection, theoretical ecology, community ecology, evolutionary biology, microbial ecology, and environmental genomics (https://www.ou.edu/ieg/seminars). The symposium, known as iFAST‐Microbial Ecology, was successfully held online in April 2022, and the majority of the members of James M. Tiedje's Academic Family were invited as speakers (https://www.ou.edu/ieg/seminars/special/ifast2204).

After the iFAST‐Microbial Ecology event, some members of Jim Tiedje's Academic Family were invited to contribute articles on diverse ecological topics in various formats to mLife and a virtual special issue on microbial ecology is assembled.

It is becoming increasingly clear that microbial diversity is extremely high in various habitats, and one of the fundamental goals in microbial ecology is to determine how such extreme microbial biodiversity is generated and maintained across space and time. The exploration of microbial processes and the scale at which they occur continues to highlight the importance of quantitative models and trait‐based models for an improved understanding of environmental biodiversity^2^ and biogeochemical processes^3^.

In the face of growing environmental challenges, Liu et al. have explored the impacts of emerging pollutants such as microplastics and nanoplastics on methanogenic digestion of waste‐activated sludge^4^. Their findings reveal the potential for resilience and functional redundancy within the digestion microbiome, despite initial suppression of methanogenesis by plastic exposure.

Climate change continues to be a pressing concern, and our understanding of its effects on microbial communities and carbon dynamics is advancing^5^, ^6^. Qiu et al. show that warming and rainfall reduction can differentially affect the abundance and composition of bacteria and fungi in semi‐arid grasslands, with implications for soil carbon efflux^7^.

One area of research that has garnered increased attention is the study of gut microbiota‐derived trimethylamine (TMA). TMA is associated with cardiometabolic disorders and represents an intriguing example of microbial involvement in the etiology of non‐communicable diseases. Comprehensive genomic screening has revealed the bacteria responsible for TMA synthesis and has led to crucial insights into their ecophysiology^8^. These findings can potentially contribute to the development of strategies to restrict TMA formation, offering a promising avenue for therapeutic intervention.

Examining the role of the mitogen‐activated protein kinase (MAPK) HOG pathway in filamentous fungi, Li et al. offer important insights into the responses of *Trichoderma guizhouense* to blue light stimuli^9^. Their genome‐wide transcriptome analysis has revealed the significant roles of the transcription factor ATF1 in regulating blue light responses, conidial germination, vegetative growth, and oxidative stress resistance. Their findings underscore the intricate interplay between light stimuli and stress signaling in fungi.

In addition to these areas of research, our fellow researchers have investigated various other aspects of microbiology, including the complex issue of species identification in prokaryotes and other microorganisms^10^, the influence of environmental selection and evolutionary processes on ecosystem functions^11^, competitive interactions within eukaryotic phytoplankton^12^, the role of microbial communities in B12 biosynthesis^13^, the effects of antibiotic resistance in the environment^14^, and the correlations between intestinal microbiota and antimicrobial resistance^15^.

We are grateful for the support and contributions of our editorial board members, authors, and reviewers, who have contributed to the success of this virtual special issue. As we look to the future, we are proud to be part of this vibrant community. This is an exciting era where we are beginning to uncover the enormous potential of unseen microorganisms. There is a pressing need to continue pushing the boundaries of our understanding and exploring the intricate relationships between these microorganisms and their environments. The study of microbial ecology is a dynamic and ever‐evolving field that continues to provide new insights about the world around us. By continuing to explore and understand microorganisms on our planet, we are not only expanding our scientific knowledge but also generating applications that will help improve the well‐being of humanity and the Earth.

## References

[1] Kamagata Y . Cultivating the unseen: lessons from James Tiedje. mLife. 2023;2:217–223. 10.1002/mlf2.12083 PMC10989887 38817816

[2] Wu L , Yang Y , Ning D , Gao Q , Yin H , Xiao N , et al. Assessing mechanisms for microbial taxa and community dynamics using process models. mLife. 2023;2:239–252. 10.1002/mlf2.12076 PMC10989933 38817815

[3] Robertson GP . Denitrification and the challenge of scaling microsite knowledge to the globe. mLife. 2023;2:229–238. 10.1002/mlf2.12080 PMC10989938 38817807

[4] Liu J , Xu G , Zhao S , He J . Resilience and functional redundancy of methanogenic digestion microbiome safeguard recovery of methanogenesis activity under the stress induced by microplastics. mLife. 2023;2:378–88. 10.1002/mlf2.12090 PMC10989149 38818270

[5] Guo X , Yuan M , Lei J , Shi Z , Zhou X , Li J , et al. Climate warming restructures seasonal dynamics of grassland soil microbial communities. mLife. 2022;1:245–256. 10.1002/mlf2.12035 PMC10989843 38818216

[6] Kuang J , Deng D , Han S , Bates CT , Ning D , Shu W , et al. Resistance potential of soil bacterial communities along a biodiversity gradient in forest ecosystems. mLife. 2022;1:399–411. 10.1002/mlf2.12042 PMC10989803 38818486

[7] Qiu Y , Zhang K , Zhao Y , Zhao Y , Wang B , Wang Y , et al. Climate warming suppresses abundant soil fungal taxa and reduces soil carbon efflux in a semi‐arid grassland. mLife. 2023;2:389–400. 10.1002/mlf2.12098 PMC10989086 38818267

[8] Vital M , Heinrich‐Sanchez Y . A small, polyphyletic group of *Firmicutes* synthesizes trimethylamine from l‐carnitine. mLife. 2023;2:267–271. 10.1002/mlf2.12079 PMC10989800 38817809

[9] Li Y , Li Y , Lu H , Sun T , Gao J , Zhang J , et al. The bZIP transcription factor ATF1 regulates blue light and oxidative stress responses in *Trichoderma guizhouense* . mLife. 2023;2:365–77. 10.1002/mlf2.12089 PMC10989065 38818272

[10] Konstantinidis KT . Sequence‐discrete species for prokaryotes and other microbes: a historical perspective and pending issues. mLife. 2023;2:341–49. 10.1002/mlf2.12088 PMC10989153 38818268

[11] Yu X , Tu Q , Liu J , Peng Y , Wang C , Xiao F , et al. Environmental selection and evolutionary process jointly shape genomic and functional profiles of mangrove rhizosphere microbiomes. mLife. 2023;2:253–266. 10.1002/mlf2.12077 PMC10989796 38817818

[12] Baker D , Godwin C , Khanam M , Burtner A , Dick G , Denef VJ . Variation in resource competition traits among *Microcystis* strains is affected by their microbiomes. mLife. 2023;2:401–15. 10.1002/mlf2.12094 PMC10989160 38818269

[13] Zhou J , Qin W , Lu X , Yang Y , Stahl D , Jiao J , et al. The diversity and ecological significance of microbial traits potentially involved in B_12_ biosynthesis in the global ocean. mLife. 2023;2:416–27. 10.1002/mlf2.12095 PMC10989127 38818271

[14] Li L , Zhang T . Roadmap to tackle antibiotic resistance in the environment under the One Health framework. mLife. 2023;2:224–228. 10.1002/mlf2.12078 PMC10989945 38817813

[15] Fu Y , Dou Q , Smalla K , Wang Y , Timothy A , Johnson TA , et al. Gut microbiota research nexus: One Health relationship between human, animal, and environmental resistomes. mLife. 2023;2:350–64. 10.1002/mlf2.12101 PMC10989101 38818274

